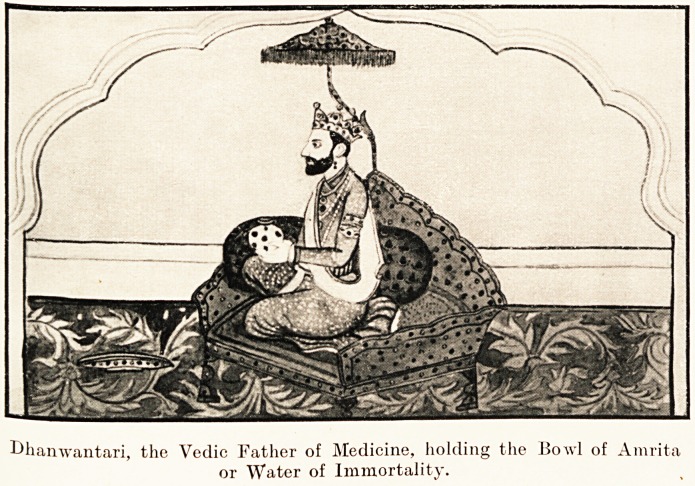# The Twenty-Sixth Long Fox Memorial Lecture: The Debt of Western Medicine to the East

**Published:** 1937

**Authors:** V. B. Green-Armytage

**Affiliations:** Gynæcologist to the West London Hospital, Hammersmith; Visiting Gynæcologist and Obstetrician to the British Postgraduate College, Hammersmith; Gynæcologist to the Hospital for Tropical Diseases, London; Late Professor of Obstetrics and Fellow of Calcutta University


### The Bristol Medico-Chirurgical Journal

*"Scire est nescire, nisi id me*
*Scire alius sciret."*

WINTER, 1937.

### THE TWENTY-SIXTH LONG FOX MEMORIAL LECTURE:

DELIVERED IN THE UNIVERSITY OF BRISTOL ON TUESDAY, OCTOBER 19th, 1937.

THE VICE-CHANCELLOR (Dr. T. LOVEDAY, M.A., LL.D.)
*in the Chair.*

BY

V. B. GREEN-ARMYTAGE, M.D., F.R.C.P., F.C.O.G.,

*Gynæcologist to the West London Hospital, Hammersmith; Visiting Gynæcologist and Obstetrician to the British Postgraduate College, Hammersmith; Gynæcologist to the Hospital for Tropical Diseases, London; Late Professor of Obstetrics and Fellow of Calcutta University.*

ON

### THE DEBT OF WESTERN MEDICINE TO THE EAST.

LITTLE did I think when I entered the portals of the Royal Infirmary thirty-five years ago that I should have the honour of being invited to deliver a lecture to commemorate the name of Edward Long Fox, a

T

man of noble character, fully imbued with that spirit of ceaseless inquiry which inspires youth.

It was not my privilege to meet him, but even at this distance of time I seem to sense his personality in the terminal words of his Presidential Address in 1894: "Our highest ambition is to leave each generation better than we found it, and by the influence of our lives to form foundations for our successors to rise still higher; to deserve to have said of each one of us, as it was said of the Great Head of our Profession, 'He went about doing good, healing all that were diseased, for God was with Him.'"

It was because of this earnestness and because he possessed an inquisitive as well as an acquisitive love of knowledge that I thought some apt tribute might be paid and some relevant inspiration gained by transporting your imagination to the Immemorial East, from whence of old the wise men came.

Hitherto it has been the pride and fashion of scholars to ascribe to the ancient Greeks the glory of having first conveyed in their own language the foundations of Medicine, Philosophy and Art; but recent research has shown that the genius of the Greeks drew its sap from Phœnicia and Crete, from Babylon and Egypt, and even still farther afield from Sumeria and the dwellers in the Valley of the Indus. Indeed, it was from the East and not from Egypt that Greece derived her architecture, her medicine, her sculpture, her science, her philosophy, her mathematical knowledge—in a word, her intellectual stimulus.

This is no idle statement; for though the Ebers papyrus carries us back a thousand years before the birth of Christ, it is only within the last fifty years that the scientific world has realized that a high state of civilization existed in Sumeria and the Indus Valley five thousand years ago.

The discoveries of Evans in Crete, of Sir Leonard Woolley at Ur, and of Sir John Marshall in Mohenjo-daro and Taxilla have illuminated our darkness, and corrected many of our former "certainties."

It has been argued by some that such discoveries are but curiosities demonstrating manual dexterity and the stimulus of environment; but if we believe, as I do, that the history of medicine is the history of civilization, and if we have seen these things as I have seen them, we shall surely agree that the real criterion of their value lies in the extent to which these ancient peoples contributed to human progress, and to that culture which is the heritage of the living world.

Woolley has shown that the earliest graves at Ur, with their wonderful furniture and ornaments of gold and lapis lazuli, are older than the first dynasty of Egypt, four thousand years before Christ, and has stated that the foundation of Egyptian civilization was due to foreign influence; for in Egypt have been found the same cylinder seals, the same architecture, the same vases, the same grotesque animal drawings, the same musical instrument (the sistrum), and above all the same religious mythology as had been familiar to the Sumerians for hundreds, may be thousands, of years.

Nor must it be forgotten that Sumerian arms and Sumerian commerce, under the ægis of Sargon, spread up the valleys of the Tigris and Euphrates, penetrating Syria, Palestine and the Taurus, carrying with them their culture and their codes, the laws of Sumer becoming the code of Babylon, the code of Hammurabi that of Moses, the code of Moses that of Israel and Greece.

Neuburger sums up our knowledge as follows: "Long before the time when Greece first appeared on the horizon of history the Babylonians were able with marvellous exactitude to undertake astronomical observations and calculations. Indeed, the style of writing, the pictorial and sculptural representation, the military tactics and jurisprudence of many nations were directly or indirectly influenced by Mesopotamia."

The code of Hammurabi (2500 B.C.), engraved on Diorite stone, regulated medical and surgical practice. Details of operations and fees are given, but the punishment meted out for unsuccess daunted and discouraged all but the very expert, with the result that surgical progress was inhibited.

Recent discoveries by Dr. Frankfort of a seal of Indian workmanship depicting the elephant, rhinoceros and fish-eating crocodile in Tell Asmar, and by Woolley, of decorated Carnelian beads and steatite vessels exactly resembling those found by Marshall in Mohenjodaro, also point strongly to the fact that so long ago as 3000 B.C. facilities of trade existed between the people of Mesopotamia and the Indus Valley. It is more than likely that the long land route was traversed, but on the other hand it is possible that the sea route was used then, as it is now, by dhows sailing from the western ports of India, hugging the shores of inhospitable Baluchistan before reaching the Persian Gulf or Aden.

In this connection it is not without interest to observe that Sir Aurel Stein has shown that the population of Baluchistan and the Indus Valley was far greater then than it is now, for in that age the climate was good. That the people of the Indus Valley traded with the whole of India, Mesopotamia and

Egypt is certain; for in the ruins of their cities we find jadeite from Central Asia, Amazon stone from the Nilgiris, gold from the Deccan, lapis lazuli and silver from Afghanistan, pearls and precious stones from Ceylon.

In those early days, though the art of writing was known, it is important to bear in mind that lack of suitable writing material made any lengthy compilation a difficult task; but modern study of folk-lore readily explains how experience, practice and learning were conveyed from mouth to mouth by the nomadic clans as they travelled along the great trade routes which stretched from India to Persia, Mesopotamia, Syria and Egypt. Those who have served in Sindh or Persia can readily visualize those long, winding caravans of mules and camels toiling and grunting along, those caravans referred to in the Books of Esther and Ezekiel carrying "ivory and ebony," "cassia and calamus," "broidered work and rich apparel" from India to the West. Is it then difficult to picture the elders of these nomads, halted at the hour of the setting sun by riverside or hamlet, telling and retelling of those wondrous things they have seen and heard? Among these assuredly the prevention and treatment of sickness must have had a foremost place.

This is no fanciful image; for medical evolution among the Chaldeans and Babylonians began some six thousand years ago, whereas among the Greeks and Romans it started some three thousand years later. The papyri of the Egyptians, the clay tablets of the Assyrians, and the Vedas of the Indians represent but an epitome of their medical achievement.

It is true that these demonstrate a sacerdotal and theurgical system of medicine, for they possessed many gods, good and evil, male and female, causing or curing disease, the ancients believing that the gods rewarded or punished man for his various acts, reward being in the form of good health and success, punishment in the form of sickness or failure.

The practitioner of medicine, whether priest or layman, was always a man of good family and education. Doubtless superstition and custom marred the palatability of his simples, but ample record exists of the variety of drugs that were in common use for everyday disorders. We read of alum, antimony, arsenic, cantharides, copper, myrrh, turpentine, mercury, iron, zinc, sulphur and fish-liver oil being employed for diseases of the eye and skin. Nor must we forget that by long observance the ancients arrived at deductions of astounding empirical value. Many of these were handed down from generation to generation. Some were observed by the Israelites, as close study of the Old Testament will show ; but perhaps the most astonishing, apart from the subject of personal hygiene and preventive medicine, is the law decreeing that man and wife shall live apart until the seventh day after the catamenial period has ceased if the people would multiply as the sands of the seashore. This empirical edict, based upon rabbinical observation, anticipates by four thousand years the recent discovery that ovulation does not occur before the thirteenth day, and perhaps also accounts for the well-authenticated fact that the orthodox Jewess does not suffer from cancer of the cervix uteri.

The Old Testament, mostly dating from eighteen hundred to five hundred years before Christ, has many references which demonstrate astute clinical observation and training. For instance, we read in Genesis xxxviii. 27 of spontaneous evolution with live uniovular twins and a ruptured amniotic sac. The treatment practised is classical and has not altered since. The condition described is very rare, only forty-four cases being on record. It was first mentioned in our medical literature by Viardel in the seventeenth century, who stated that when twins are of the same sex they are enclosed in a single amnion, whereas when they are of different sexes the Almighty encloses them in separate sacs with a view, shall we say, to guarding their morals *in utero!* Then again we have recognized the extreme dangers of uterine inertia in 2 Kings xix. 3 and in Jeremiah xv. 19, together with the fatality associated with precipitate labour and inversion or postpartum hæmorrhage in 1 Samuel iv. 19. I hope some of you will refer to these passages. But perhaps most wonderful of all is the graphic description (far antedating Hippocrates) of puerperal or pelvic peritonitis in Numbers v. 21–27.

Obstructed midwifery has little or no mention, but that is what one would perhaps expect in a nomad people living a healthy life and feeding on a full vitamin diet. The words "the obstetric hand shall bring forth the winding serpent," in Job xxvi. 13, would, however, seem to hint at version or manual removal of the placenta.

Remember always that the treatment of disease in Biblical times was not *only* by prayer and sacrifice, for dietetic measures and medicaments were used, including healing springs and oil baths. Oil, wine and balsam were employed in dressing wounds, and bandages were applied to broken limbs. Midwives were obviously esteemed, for we read that "the Lord dealt well with the midwives and built them houses."

Though the priests or Levites were health officers, it is wrong to suppose that no professional physicians existed ; for wherever there is mention of healing there is no reference to priests, but rather to the " Rophe " or physician. It will be recalled that Jeremiah held it as inconceivable that there should be no physicians in Gilead, and of course the testimony of Ecclesiasticus xxxviii. stands for all times.

The Talmud gives much useful information, and shows considerable knowledge of the dura and arachnoid membranes. The preparation of food from slaughtered animals gave the Jews insight into the pathology of diseases of the liver, lung, kidneys, etc. Moreover, we read that trephining, amputation and venesection were regularly performed.

It would be indeed erroneous to minimise the medical achievements of the Babylonians and Israelites twenty to thirty centuries before Christ merely because we possess no doctrinal evidence. Admittedly magic, astrology and divination befog the written word, but there can be no question that the practice of embalming, together with clinical observation, advanced the knowledge of anatomy, physiology and surgery, so that succeeding generations of Greeks and Romans could build upon it, assisted by speculative philosophy and deductive observation. Should such a statement be challenged one has only to contemplate that masterpiece of Assyrian art, the dying lioness from the palace of Assurbanipal which is in the British Museum. The wounded beast, her spine severed by an arrow, turns snarling on the hunters, dragging her paralysed limbs. Such a conception could only be depicted by an artist fully acquainted with the physiological effects of spinal injuries.

It is not possible to date the dawn of Indian civilization, but it is probable that the Aryan race, fair in complexion and enterprising in character, originated in the high tableland eastward of the Caspian Sea. That they possessed a co-ordinate system of government and a religion abounding in moral precepts is certain. As their numbers increased and expansion became necessary one branch spread westwards towards Persia and the Caucasus, the other spread south-eastwards, invading and settling in a country called Aryavarta (the land of the Aryans), which lay east of the River Sindha, the western boundary of India. The letters H and S are convertible in Zend, the ancient Persian language, and so the name of the River Sindha explains the title given by those who first passed its boundaries—the Hindus.

The Book of Kings in our Old Testament speaks of the wisdom of a people who inhabited the east of Asia, and in Genesis xxxvii. 25 we read of the products which were in demand from them. Moreover, we are told that Terah, the father of Abraham, who lived about 1800 B.C., "dwelt on the other side of the flood and served other gods," the flood, of course, denoting the great Euphrates River.

The Aryan race, possessing both an alphabet and grammar, were the founders of Sanskrit, the purest and most perfect of all languages, from which Zend, Armenian, Greek, Latin, German and Celtic derive their basic origin.

At this point it is not without interest, perhaps, to observe that all the early religions, those of Brahma, the Buddha, Confucius, Mahomet and Christ, together with almost all early civilizations, such as those of Sumeria, Northern India, Babylon, Egypt, Crete and Greece, flourished round about the thirty-fifth degree of latitude; that is to say, where the resources of nature were most abundant and the climate not too enervating.

It has been supposed by some that China can claim priority in knowledge of the arts and sciences owing to the remarkable and enduring character of their civilization, but although there was intimate intercourse with India by means of travellers, ambassadors and Buddhist priests, it is now held that the science of medicine was not introduced into China before the Christian era, for reverence of the dead and ancestor worship forbade dissection. The only medical debt we consciously owe to the Chinese is gold and silver acupuncture, which was immemorial treatment for every ill ; though it would be churlish not to admit that they first discovered the art of making paper, an art that later made the intellectual revival of Europe possible.

It is from the Vedas, through the mists of mythology and fogs of superstition, that fifteen hundred years before Christ we can discern the realities of existence in ancient Hindustan. Legend has it that the great god Brahma wrote the Vedas for the guidance of the universe, and that the greatest of these was the Atharva Veda. Subsequently, taking compassion upon sick and suffering man, Brahma produced the Ayur Veda, which is the first great treatise on the Science of Life. Dhanwantari, the father of medicine, was then created to minister to the diseases of mankind, and just as the sages came in later centuries to Hippocrates, so in those early days deputations were sent to Dhanwantari for instruction. The story runs that on their arrival the master asked his pupils, " On what shall I lecture ? " and they answered, " On surgery, because among the gods there are no diseases, whereas wounds and injuries are the common lot of gods and

### Ancient Hindu Surgical Instruments.

Wolf Skull Forceps — Curlew Ear Forceps — Cleft Palate Knife — Ancient Hindu Cataract Knife

Crocodile Bone Forceps — Tiger Bone Forceps — Ancient Hindu Long Toothed Forceps

Lion Bone Forceps — Ancient Hindu Ear Forceps — Ancient Hindu Trocars and Cannulae

Ancient Hindu Bone Forceps — Ancient Hindu Cautery

Dhanwantari, the Vedic Father of Medicine, holding the Bowl of Amrita or Water of Immortality.

men." This reply, so like the story in Homer of Achilles and Machaon, is not without interest, for even to-day many incline to look upon disease in mankind as a retribution for sin.

Susruta and Charaka were among the first disciples of the master, and though some are of the opinion that their Sanskrit compilations belong to the Christian era, it is far more probable that they are pre-Hippocratian, for their elaborate ethical code, written in ancient and purest Sanskrit, was founded on the Laws of Manu, and is purely Brahmanical. These laws date back to 900 B.C. Moreover, a study of their copious Materia Medica reveals the fact that there is not a single mention of any substance of foreign source, whereas the writings of Hippocrates give ample evidence that many Indian plants, whose properties were well known, were imported into Greece.

The Hippocratic oath leads one to the same conclusion, for when it interdicts the performance of lithotomy, except by men who were special practitioners of this operation, it surely suggests that the author's knowledge of anatomy and surgery had been derived from a more enlightened nation, and was imperfect compared with that of his teachers. Moreover, seeing that Hippocrates visited Smyrna, Libya and Scythia, is it not probable that he visited this latter distant and inhospitable northern country because he had heard of the surgical feats performed by an enlightened people ?

Dim dates are difficult to be sure of, but at least we know that at the time of India's invasion by Alexander the Great the conqueror enlisted the services of local practitioners, whose wisdom was as proverbial then as it was in the later days of Galen,

Pliny and Dioscorides, who frequently when wishing to imply excellence or wisdom use the words *Indi dixerunt.*

Susruta taught that the foundation of surgery was anatomy, and made his pupils do dissections. In those days there was no Brahmin ban against touching a corpse, for the Laws of Manu state that "mere bathing will purify after touching a dead body, while to stroke a cow or gaze at the sun if the mouth be sprinkled with water will remove defilement."

Thus began the golden age of Hindu surgery. To Susruta we owe the discovery of cataract-couching, skin-grafting and rhinoplasty. From him we possess a precise knowledge of midwifery and learn the positions occupied by the fœtus *in utero.* He states that the expectant mother must be kept in a happy frame of mind and in placid surroundings if her labour is to be easy. He speaks of post-mortem Cæsarean section. He writes of amputations and the necessity of artificial limbs made of iron. Tumours are removed, ruptures reduced and patients cut for stone. Still more remarkable, rules are laid down for the operating-room, for it is written that it should be fumigated with sweet vapours, the surgeon is to keep his hair and beard short, his nails clean and wear a sweet-smelling dress. It is not certain what drug was used, but directions are given that the patient was to inhale a substance called *sammohini* before operation. Over a hundred steel instruments are depicted and their use described. Many of these have their counterparts in the catalogues of every modern firm supplying the needs of the general surgeon.

Reference having been made to the fact that Cæsarean section is mentioned by Susruta, perhaps a digression may be permitted in order to show that this operation is of great antiquity, indeed dating back to the Iliad of Homer; for the legend runs that Coronis, mother of the unborn Aesculapius, was killed by Artemis for her infidelity. When Apollo, the father of her child, saw Coronis on the funeral pyre, he cut the boy from his mother's womb and carried him to the cave of the wise centaur Chiron, where he was instructed in the cure of all disease.

Ovid, *Metamorphoses* ii. 630, obviously refers to this belief in the lines :—

> "Natum flammis uteroque parentis
> Eripuit geminique telit Chironis in antrum."

And, of course, we have the evidence of Shakespeare that the operation was well known in his day, for apart from the reference in *Macbeth* to "Macduff who was from his mother's womb untimely ripped" we have the metaphor in *King John*, V. iii., indicating that his audience was well acquainted with the operation :—

> "You bloody Neros, ripping up the womb,
> of your dear Mother England, blush for shame."

There can be no question that the practice of Cæsarean section dates back to the Vedas, for to-day, as from times immemorial, the Hindu is forbidden to burn the body of a pregnant woman who dies before the birth of the child without first excising the fœtus.

Needless to say such evisceration must have taught the elements of anatomy; for the most frequent cause of premature death in the case of a pregnant woman in the Tropics must have been the same then as now, namely either enormous enlargement of the spleen and liver inhibiting progressive ascent of the uterus or obstruction in the pelvis preventing delivery.

Charaka flourished about 400 B.C. and wrote eight books. He describes fevers, leprosy, mania, epilepsy, and nearly every disease we meet with in common practice to-day. The seventh book details four hundred purgatives alone and their various uses. He classifies food and diet according to disease, and tries, like Freud, to analyse the secret of dreams. He writes of the benefit of steam baths and of stramonium cigarette smoking for asthma. He advocates nux vomica for paralysis and dyspepsia. There is an interesting section on climatology, where patients, according to their sufferings, are urged to sojourn in such climates as are suitable to them. The ritual of oral cleanliness is looked upon as of the first importance, and although toothbrushes did not come into use in Europe until about A.D. 1700, it is remarkable to read the rules laid down by Charaka for the selection and use of twelve distinct types of toothbrush and tooth powder.

He tells us that the practitioner who knows the value of quicksilver and the metals is a god, he who knows the qualities of herbs and roots is a man, he who knows the use of the knife or fire resembles a devil, but he who knows the proper prayers to be offered up in the time of sickness is a prophet.

The mention of the metals is of significance, for the ancient Hindu race were the first to employ mineral drugs internally. Their chemical skill was remarkable. They knew how to prepare sulphuric, nitric and hydrochloric acid. Gold, mercury, silver, copper, lead, tin, zinc, iron, arsenic and antimony are all described, and their uses, either in pure form or as salts, are indicated. For instance, it is illuminating to read that if sixteen parts of gold and one of lead be mixed with lemon juice and then rubbed together and heated, or if gold be mixed with sulphur and aloes and exposed to the fire twelve times until it is reduced to a powder, either mixture is valuable for chronic diseases of the spleen and lung. Or, again, it is instructive to read of the preparation of the yellow sulphurate of arsenic, which was used for leprosy, skin diseases, mania and fevers.

It would take too long to recount the way they prescribed their medicines in pill, extract, infusion or ointment form, with rules which varied according to the temper, age, constitution and disease of the patient, but historically it is perhaps not without interest to see that medicines were divided into classes, one which increased strength by evacuating bad humours from the body, such as purgatives and emetics, and the other which lowered the exalted action of the humours and restored them to a healthy state. For instance, thirty-nine simple drugs are enumerated for curing diseased wind, twenty-three for curing diseased phlegm and twenty for deranged bile. In those days rubbing with oil to relax all tissues of the body was an essential feature of treatment before any drugs were exhibited.

In parenthesis and lighter mood it may not be without interest to the gentler sex to relate that the art of dyeing the hair, the fashion of lipstick and eye pencilling, the mode of varnishing the finger and toe nails, together with the music of the bagpipes, all originated in Sumeria and the north-west corner of India some five thousand years ago—imperishable legacies to many.

If such was the wisdom of Aryan India (and I have but given you a passing glimpse of it), what was the reason of its decay ? History tells us that it was the rise of pure Brahmanism with the desire of the priests to maintain all power in their own hands. For it must be remembered that the Brahmins, with their subcaste the Vaidyas, considered themselves persons of divine origin, whose duty it was to perform rites of religion and instruct mankind in the path of learning and duty. They alone had access to the Shastra, and so became learned pundits and skilful physicians.

The priestly office, like that of the ancient Druids, was the road to distinction and power. In the beginning they attained pre-eminence by cultivation of the mind and the practice of virtue, but with power came degeneracy. The despotism of caste, forbidding liberty of thought, laid down rules which subjected a whole continent; for we see that in order to curtail the growing influence of the surgeon they not only prohibited all shedding of blood, but ruled that all who took from or were touched by a surgeon became unclean, and to enhance their own majesty they introduced the treatment of all diseases by *mantras,* incantations and amulets.

The rise of Buddhism and its rapid spread throughout the Far East still further inhibited surgical progress for hundreds of years, for the teaching of the Buddha prohibited the use of the knife and any form of dissection.

One must, however, be fair to the Buddhists, for to them India and the whole world owes a great debt from a sanitary, medical and veterinary point of view. They first established a system of state medicine, one physician being appointed for every ten villages on the great roads of India. They laid down laws regulating burial and sanitation. They prohibited adulteration of food. They established botanical gardens for the special supply of herbs and drugs for medical use. They built bird and animal sanctuaries and even animal hospitals with a reverence worthy of St. Francis of Assisi. Indeed, our debt to the followers of the Buddha is but little appreciated, and I would wish to offer them a professional salute.

It is not easy for the Western mind to enter into that spirit of Buddhist or Hindu philosophy, which deprecates action, for, as Carlyle has said, "the end of man is an action and not a thought, though it were the noblest."

The Yogi who practises Yoga with the object of uniting his soul with the divine spirit is highly interesting as an example of the attitude of the Indian mind towards life. His world-weariness causes him to fly from the struggle and pleasures of life to the solitude of the jungle, in order to seek an escape from the activity even of thought itself.

It may be that a philosophy of quietism was natural to the indolence and enervation of Indian life, but such a doctrine was a direct incentive to warlike and materialistic neighbours to harass and invade the country; and so we find that all the learning of centuries subsided, was lost or forgotten in the tides of war and invasion which swept again and again over India from the beginning of the Christian era.

Is it then surprising that the clocks of medicine and of surgery stood still, and that Europeans visiting Hindustan at the time of Shah Jehan marvelled at the lack of any surgical skill? Their wonder is perhaps not so remarkable when one considers that the doctrine of Karma held two hundred million people, a doctrine that taught that "I am what I am because of my past deeds, I shall be what I shall be because of my present deeds."

So far it has been my object to portray the intellectual vigour of the primitive eastern branch of Aryans, to whom we owe so much in medicine, law, art and philosophy. It is not my intention to dwell upon that later western branch which built up the Grecian and Roman systems of medicine from the time of Pythagoras till the second century A.D. During this period men of genius conferred glory on their country and imperishable renown upon themselves. Greece became the seat of philosophy, and Greeks the nursemaids of the arts handed down to them by earlier eastern civilizations. But as in India so too in Greece, the clock of progress was stopped by the invasion of rude and hardy races from the north, who destroyed what was most noble and beautiful with relentless barbarism, preserving only what harmonized with their rude manners or administered to their sensual pleasures. Thus persecuted, Europe remained for many centuries in a state of lethargy, the pendulum of learning swinging back again towards Egypt and Syria.

It is to the Caliphs of Baghdad that we owe an imperishable debt. They patronized learning and instigated the translation into Arabic of the most important medical authors, particularly those of Hippocrates, Dioscorides, Galen and Paul of Aegina. This inheritance of Grecian thought awakened the dormant intelligence of the Arabs and sharpened their critical faculties. The Nestorian school at Jondisabur was the cradle of Arabic medicine. From it came wise men who gave the impetus to translation and scientific research which lasted for centuries. To fulfil this statement it is only necessary to mention three great names, Rhazes, Ali Abbas and Avicenna.

Rhazes was born in 850 A.D. He was guided by the principles of Hippocrates, basing his opinions upon observation and laying stress upon hygienic and dietetic measures. He has acquired lasting fame chiefly for his vivid description of smallpox and measles.

Ali Abbas realized that the chief task of the physician was to prevent disease. He was a pioneer in recommending that young physicians should frequent the mental and other hospitals which were first inaugurated by and were the glory of Arabic civilization from Baghdad to Alexandria and from Cairo to Cordova.

Avicenna, who wrote the *Canon of Medicine,* stands out as a monumental figure from the tenth to the fifteenth century. He was a statesman, physician, astronomer, teacher and author. His great work is a landmark, for it stands as an epitome of all precedent development, the final codification of all Græco-Arabic medicine.

For nearly a thousand years Arabic physicians were the teachers of the West, throwing open to the Christians of Europe the portals of scientific medicine and granting to them the privilege of access to the intellectual treasures of antiquity, a privilege of surpassing importance which compels recognition.

With the Renaissance individuals and nations vied with each other in honouring the pursuit of learning. The study of medicine exercised the genius of great men, who constructed it upon a basis of anatomy and physiology, observation and reasoning, not a little assisted by the genius of Leonardo da Vinci, who uplifted the science of descriptive and pictorial anatomy to a height which has never been equalled.

Coming to more modern times, it is very easy to see and appreciate the debt of the West to the East, for civilization, commerce, transport, peace and war, owe an imperishable debt to scientists working under conditions of danger and stress.

If the apothegm of Blaise Pascal, "Had the nose of Cleopatra been shorter, the whole face of the earth would have been changed," be accepted, how much more truly could it be written, "Had the cause and treatment of malaria been known, to what heights would not Greek and Roman culture have reached."

History teaches us that Hippocrates recognized the periodicity of malaria and relapsing fever, for he describes the jaundice, the enlarged spleen and liability to abortion; but it was the tide and pride of wars, with overcrowding of populations in infected areas, that spelt the ultimate decay of Greece and Rome.

The story of Ronald Ross working in Calcutta before the days of modern comforts and modern scientific implements is a romance in itself. It is quite impossible to estimate the benefits that his discoveries and those of Manson have conferred upon mankind—discoveries that have made possible the development of all tropical countries, and which have reduced the death-rate of immigrants and indigenous people in those countries to that of London.

Similarly peace and war have demonstrated the debt we owe to the experimental research of Shiga and Leonard Rogers on the treatment of dysentery and cholera; to the work of Obermeyer, Todd and Mackie on the transmission of relapsing fever; to the discoveries by the Japanese investigators Inada and Ido of the leptospiræ which cause Weil's disease; to the researches of British, Dutch, German and Indian workers on the causation of beri-beri and epidemic dropsy ; to the work of McCarrison on goitre and nutrition ; to the finding by Bilharz of the parasite which, since time immemorial, has been the cause of colossal mortality and invalidism ; to the investigations of Scott, Fairley and Mackie on the ætiology of sprue, that disease which so often in former days was fatal ; to the work of Leishman and Donovan upon kala azar ; and to the elucidation of that crippling disease osteomalacia by Maxwell, Stapleton and others. It is not necessary to prolong the list, for these are but a few of the diseases which every European practitioner may meet with in these days of rapid transit and moving populations.

Horace tells us that as we grow older we become *mitior et melior ;* therefore, before closing, I should like to touch upon a somewhat sombre side of our debt, one that has a bearing upon the future. Hitherto I have endeavoured to demonstrate what we owe to the East, and have tried to illustrate how, in this century at least, we have repaid some of that debt in the currency of preventive medicine. But from that currency an imponderable problem is arising, namely that of food production and supply, for a rapidly increasing eastern population with a rapidly decreasing death - rate. The burden that preventive medicine has thrown upon the East is an immense one. It is a challenge, for starvation and ultimate decay must occur if food production is inadequate in those countries. It is for us to devise a plan lest the debt of eastern medicine to the West be repaid by lamentation.

That science will solve the problem, too, we need not doubt. The productivity of Mother Earth is so great that even in a crowded Europe and America millions of tons of fresh foodstuffs are destroyed deliberately every year for purely economic reasons. "Poverty amid Plenty" is a commonplace all the world over. But it is a problem which we must solve quickly, especially in the East. If we can solve it, maybe our debt to those "Wise Men of the East" of whom I have been speaking will be *at last* repaid in full.

## Figures and Tables

**Figure f1:**
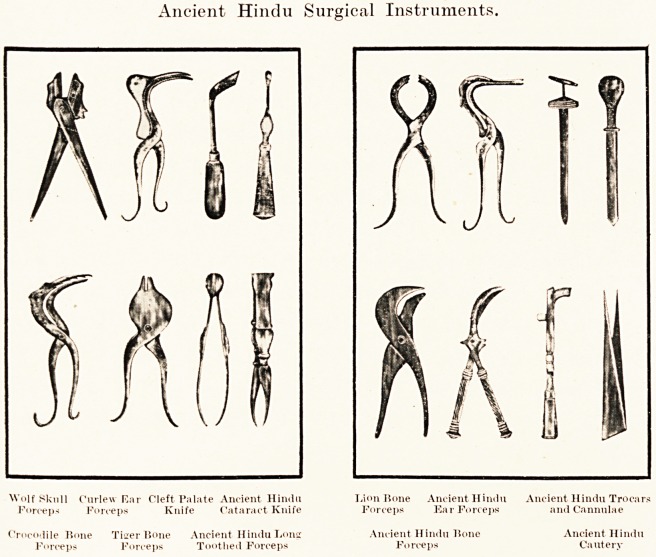


**Figure f2:**